# Vulnerabilities in the Tau Network and the Role of Ultrasensitive Points in Tau Pathophysiology

**DOI:** 10.1371/journal.pcbi.1000997

**Published:** 2010-11-11

**Authors:** Theresa M. Yuraszeck, Pierre Neveu, Maria Rodriguez-Fernandez, Anne Robinson, Kenneth S. Kosik, Francis J. Doyle

**Affiliations:** 1 Department of Chemical Engineering, University of California, Santa Barbara, Santa Barbara, California, United States of America; 2 Kavli Institute for Theoretical Physics, University of California, Santa Barbara, Santa Barbara, California, United States of America; 3 Neuroscience Research Institute, University of California, Santa Barbara, Santa Barbara, California, United States of America; 4 Process Engineering Group, IIM-CSIC, Spanish Council for Scientific Research, Vigo, Spain; 5 Department of Chemical Engineering, University of Delaware, Newark, Delaware, United States of America; 6 Department of Molecular, Cellular, and Developmental Biology, University of California, Santa Barbara, Santa Barbara, California, United States of America; 7 Institute for Collaborative Biotechnologies, University of California, Santa Barbara, Santa Barbara, California, United States of America; Washington University in Saint Louis, United States of America

## Abstract

The multifactorial nature of disease motivates the use of systems-level analyses to understand their pathology. We used a systems biology approach to study tau aggregation, one of the hallmark features of Alzheimer's disease. A mathematical model was constructed to capture the current state of knowledge concerning tau's behavior and interactions in cells. The model was implemented *in silico* in the form of ordinary differential equations. The identifiability of the model was assessed and parameters were estimated to generate two cellular states: a population of solutions that corresponds to normal tau homeostasis and a population of solutions that displays aggregation-prone behavior. The model of normal tau homeostasis was robust to perturbations, and disturbances in multiple processes were required to achieve an aggregation-prone state. The aggregation-prone state was ultrasensitive to perturbations in diverse subsets of networks. Tau aggregation requires that multiple cellular parameters are set coordinately to a set of values that drive pathological assembly of tau. This model provides a foundation on which to build and increase our understanding of the series of events that lead to tau aggregation and may ultimately be used to identify critical intervention points that can direct the cell away from tau aggregation to aid in the treatment of tau-mediated (or related) aggregation diseases including Alzheimer's.

## Author Summary

Neurodegenerative disorders, particularly the tauopathy Alzheimer's disease, affect millions of people and cost billions of dollars a year in healthcare costs. Although effective treatments to delay or reverse cognitive decline are still unavailable, several approaches to address this medical need are being pursued. One such strategy involves ameliorating aberrant tau processing, as the characteristic tau tangles associated with the tauopathies are well-correlated with cognitive dysfunction, genetic mutations in tau lead directly to neurodegeneration, and experiments in animal models have yielded promising results. Two avenues are currently being explored: inhibition of kinase activity to reduce the presence of aberrant, hyperphosphorylated tau and means to prevent and reduce tau aggregation. We have taken a systems biology approach to understanding tau pathophysiology, creating a mathematical model to quantitatively explore the vulnerabilities in the tau network and identify effective intervention points. Our analysis of the resulting *in silico* neuron populations, representing healthy and aggregation-prone neurons, highlights the multifactorial nature of the disease and provides insight into pathological triggers and the timing of treatment, which will be an important element in effectively treating patients.

## Introduction

Despite the fidelity of protein folding and the operation of quality control mechanisms to eliminate misfolded and otherwise abnormal proteins, a number of diseases can be traced to defects in these processes [1]. Among them are many neurodegenerative disorders, including the tauopathies, which are characterized by the intraneuronal aggregation of tau protein and of which Alzheimer's disease (AD) is an example. Preventing aggregation to halt or reverse cognitive decline is the goal of many drug discovery programs, but effective, long-term treatments have yet to be discovered [2].

A convincing body of evidence implicates defective tau processing and the formation of intraneuronal tau aggregates in cognitive decline. Mutations in the gene encoding tau protein are directly responsible for a number of genetic conditions collectively called primary tauopathies, among which is frontotemporal dementia and Parkinsonism linked to chromosome 17 (FTDP-17) [3], [4]. Tau pathology is also present in a large number of conditions whose cause cannot be traced to mutations in the gene encoding tau, including traumatic brain injury and repeated head trauma (dementia pugilista) from contact sports [5]–[7] as well as Alzheimer's disease, and has been observed with and without amyloid-beta pathology. Post-mortem assessment of the neurofibrillary tangle load in the brains of demented human patients showed that the severity of dementia was well correlated with the presence of tangles, a finding that argues strongly that tau plays a central role in disease progression [8]–[10]. In addition, the deficits in spatial learning and memory observed in mouse models expressing human APP can be ameliorated by reducing endogenous, wild-type tau [11], which also protects against early mortality and inhibits excitoxicity; this finding is supported by more recent experiments in an AB-forming mouse model [12]. Taken together, these studies point to tau as a key causative factor in neurodegeneration and suggest that the tau pathway itself represents a reasonable therapeutic target for diseases in which the abnormal tau processing pathway is triggered.

Tau is a neuronal, microtubule-associated protein (MAP) whose physiological function is to regulate microtubule dynamics (Figure S1). Alternative mRNA splicing yields 6 protein isoforms that are divided into two broad classes according to whether they contain 3 or 4 microtubule binding repeats; they are known as the 3R and 4R isoforms, respectively [13], [14]. The 4R isoforms have a higher affinity for microtubules and greater tendency to aggregate [15]–[18]. A phospho-protein with nearly 30 phosphorylation sites, tau's biological activity is also governed by its phosphorylation state. In a healthy neuron, tau contains 2–3 moles of phosphate per mole of tau and is found almost entirely bound to microtubules [19]. In degenerating neurons, kinase and phosphatase activity is dysregulated and an abnormal variant containing 5–9 mol phosphate/mol tau is generated. While normal amounts of physiological tau are maintained, high amounts of hyper- and abnormally phosphorylated tau with low affinity for microtubules and resistance to degradation are generated [20]. These tau species dissociate from microtubules and collect in the cytosol, where they subsequently misfold and aggregate. The presence of ubiquitin, a molecular tag that facilitates degradation by the proteasome, in the aggregates suggests a failure of the quality control systems that clear aberrant proteins, contribute to the accumulation of abnormal tau and the neurofibrillary tangles [21]. Experiments demonstrating that the ubiquitous, constitutively expressed chaperone Hsc70 binds tau support this view, as Hsc70 is a chaperone known to mediate a protein triage decision that results in either refolding or degradation [22]. When the cell's quality control systems fail, tau aggregates and eventually neuron death occurs. The long, insoluble filaments that form may serve as a ‘stop-gap’ measure to protect the cell from adverse consequences by sequestering toxic intermediates. However, the actual toxic moiety among various pathological tau states has not been conclusively determined.

The multifactorial nature of disease motivates our systems biology approach to understanding tau pathophysiology. We have developed a computational model that represents the network of interactions in which tau is involved as a system of ordinary differential equations that describe the deterministic chemical kinetics. The model was tuned to capture observed behavior in a healthy neuron and an aggregation-prone neuron. Although the class of tauopathies contains several diseases, specific experimental data from Alzheimer's disease studies informed this model. Sensitivity analysis tools were used to interrogate the model and ascertain the relative contributions of each component in the tau pathway from its synthesis to its post-translational modifications, to its degradation. Within both populations of neurons, and particularly the aggregation-prone population, we found ultrasensitive cellular conditions that are likely to be resistant to rescue.

## Results

### Model structure

**Figure 1:**
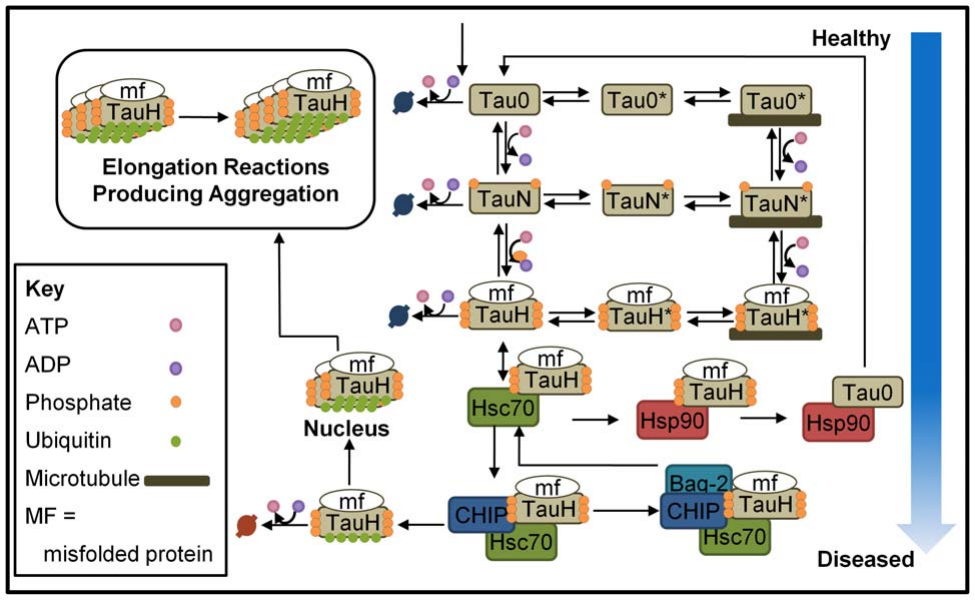
Model structure of tau pathophysiology for a single isoform. The network captures tau phosphorylation and dephosphorylation, microtubule binding and release, uptake, rescue, and degradation by the chaperone machinery, and aggregation. Specifically, unphosphorylated tau (Tau0) can be degraded or phosphorylated, producing normally phosphorylated tau (TauN). TauN can also be degraded in a non-ubiquitin dependent fashion, dephosphorylated, or phosphorylated to create abnormal tau (TauH), which can likewise be degraded, dephosphorylated, or phosphorylated. Each of these free tau species undergoes a conformation change to produce a form with high affinity for microtubules; these species are denoted with a star as Tau0*, TauN*, and TauH*. Abnormal TauH is taken up by the chaperone Hsp70, which mediates the decision between rescue and degradation. Both isoforms participate in the same series of reactions, but at different rates of reaction, and their behavior is coupled through the chaperone and degradation machinery.

As one of the first attempts at *in silico* simulation of tau pathophysiology, a mathematical model representative of the known biology was established within the limitations of the available data (Figure 1). Although this model is necessarily a simplified version of reality, it captures essential features of the known tau network and could be easily extended to incorporate additional detail as new data is generated. Among the key components are the 3R and 4R isoforms of tau. Alternative splicing of other tau exons was not considered in the model; therefore we modeled two species to be representative of the 3R and 4R classes. The isoform classes were divided into a number of phospho-states; although there are likely many disease-relevant phospho-isoforms, for simplicity, each 3R and 4R form was divided into in a minimally phosphorylated, normally phosphorylated, or abnormally phosphorylated/conformationally altered state. Minimally phosphorylated 3R and 4R tau are constitutively produced in a single reaction that captures transcription and translation. Specific tau kinases and phosphatases such as GSK3-β and PP5A were not explicitly included in the model. The kinetics of phospho-isoform conversion were modeled using Michaelis-Menten kinetics and based on *in vivo* data, from which the bounds on the Michaelis-Menten constants and the dependence of the kinetics on the phospho-state were derived. Tubulin, the building block of microtubules, was included although the total pool of tubulin with which tau interacts was considered constant throughout these analyses. Makrides and colleagues [23] monitored the *in vitro* reaction kinetics between tau and pre-assembled microtubules and found that a two-step mechanism in which either tau or tubulin underwent a conformational change before binding fit the data best; we employed that two-step mechanism here, assuming the conformational change occurred in the tau protein prior to association. Tau degradation by the proteasome has been shown both *in vitro* and *in vivo* in neuronal cell culture [24], and has also been shown that natively unfolded tau can be degraded by the 20S proteasome in a non-ubiquitin dependent manner [25]. This degradation process was modeled with first order kinetics and a constant pool of proteasomes.

Abnormal 3R and 4R tau are bound by the chaperone Hsc70 [22], which mediates a choice between rescue and ubiquitin-dependent degradation. We assumed a simple, reversible binding reaction that does not involve ATP; although Hsp70 is usually an ATP-dependent chaperone, recent evidence suggests it binds tau independently of ATP [22]. Rescue is facilitated by the chaperone Hsp90 [26], [27]; although other proteins such as the peptidyl-prolyl isomerase PIN1 are likely to participate in this pathway [28], we assumed a simple mechanism by which Hsp90 binds abnormal tau. In this simplification, abnormal tau is dephosphorylated and restored to its normal functional form upon Hsp90 binding, and is released to re-bind microtubules. CHIP, an Hsc70-interacting protein and E3 ligase, links the chaperone and degradation machineries and shuttles abnormal tau to the 26S proteasome [29], [30]. BAG-2 binds with the CHIP-Hsc70-Tau complex and subsequently dissociates with CHIP, restoring the Hsc70-Tau complex B, acting to potentially rescue tau from CHIP-mediated degradation [31], [32]. Alternatively, CHIP and Hsc70 can release ubiquitinated, abnormal tau in a single-step reaction, after which tau is degraded. Because tau has been shown to be abnormally phosphorylated prior to ubiquitination, we assumed that only the abnormal tau species could be degraded in a ubiquitin-dependent, chaperone-assisted manner [33]. Aggregation is an alternate pathway down which abnormal tau can travel. Tau aggregation was modeled with the nucleation-elongation reaction mechanism and kinetics established by Congdon et. al. They monitored *in vitro* tau fibrillization and found that a tau dimer acted as the nucleus for the reaction, best fitting the experimental data and providing a good prediction of the length distribution of aggregates through time [34]. We assumed that only abnormal, ubiquitinated tau could polymerize as the presence of ubiquitin in tau aggregates is well-established [35], [36] and full-length, wild-type tau does not aggregate readily under physiological conditions *in vitro* in the absence of polymerization promoters because it is hydrophilic and relatively unstructured [18]. Although normal tau may be sequestered by abnormal tau and thus aggregate [37], this mechanism was excluded from our construction due to a paucity of available data. Furthermore, the paired helical filaments into which abnormal tau aggregates in Alzheimer's disease patients contain 3–4 times more phosphate than physiological tau and the level of phosphorylation observed in soluble amorphous tau is similarly elevated, suggesting that paired helical filaments are primarily comprised of abnormal tau [19], [38]. In a study of brains from patients diagnosed with the tauopathy FTDP-17, in whom tau is mutated, the insoluble fraction was observed to have a much greater ratio of mutated tau than normal tau [39], also supporting this assumption. The effect of macromolecular crowding was also neglected for parsimony. Excluding these mechanisms from our model is likely to have little effect on the qualitative results, resulting in a re-scaling of parameters but not substantially changing the qualitative behavior and overall conclusions.

Mass action kinetics described all reactions in the network except the phosphorylation and dephosphorylation reactions, which were described by Michaelis-Menten kinetics. For each species represented by our model, an ordinary differential equation that describes the species time-evolution was constructed as illustrated in Eq. S1. In total, the network contains 84 reactions, 93 parameters, and 45 states (i.e., differential equations). A full listing of the states, reactions, parameters, and differential equations can be found in Tables S1 and S2.

### *A priori* identifiability

Parameter space for the healthy and aggregation-prone identifiability and optimization steps is different, as the chaperone and degradation machinery was considered to be operating homeostatically. As a result, before initiating each stage of the optimization, an *a priori* identifiability analysis was completed. Correlation matrices were calculated at 1024 quasi-random points in the relevant parameter space, each matrix was weighted based on the objective function value determined at its corresponding location in parameter space, and then the matrices were averaged to establish pseudo-global *a priori* identifiability.

The results of both stages of this analysis confirm that the proposed model is *a priori* identifiable and, by extension, structurally identifiable (Figures S2 and S3). To improve the efficiency of the optimizations, we did remove three parameters (k_1_, k_84_, k_10_) from the first stage of the procedure as they were highly correlated (>0.95).

### Establishing healthy neuron models

In the next step, we optimized parameters to achieve steady-state behavior that represents healthy neuron function. Parameters associated with phosphorylation and dephosphorylation, microtubule binding and release, synthesis, and ubiquitin-independent degradation were estimated. We also estimated ATP synthesis and depletion. Parameters were generally assumed isoform-independent, with the exception of the microtubule binding parameters and aggregation parameters. Because evidence suggests that 4R tau has a greater affinity for microtubules [15]–[17] and for aggregation, these parameters were increased relative to the corresponding reactions involving 3R tau. Estimating chaperone and degradation parameters was excluded from the healthy state computations because under normal conditions Hsc70 does not bind microtubule bound tau [22]. Although Hsc70 may bind free normal tau species, these species represent a small portion of total tau and thus the model was simplified to exclude these minor interactions.

The objective function that mathematically quantifies the behavior of a healthy neuron was constructed to reflect known quantitative experimental data. It is well-established that aberrant tau species are undetectable in normal neurons; thus we require that free and microtubule-bound aberrant tau is minimized. From measurement of total tau in human brain homogenates [40], and assuming total protein concentration is 500 mg/ml [41], the total neuronal concentration of tau protein was estimated to be 5–10 µM, consistent with many reported values. In adult human brain that is not afflicted by Alzheimer's, the ratio of 3R to 4R tau was determined to be 1:1 [14], [42]. The affinity of normal tau for microtubules is 16 nM [23] and at least 80% of the total neuronal tau is bound to microtubules. These data are quantified in a cost function that sums the squared percent difference between the model result and the experimental results. Several of the objectives in our cost function are “fuzzy”, i.e. they allow states to achieve a range of values without penalty, rather than admitting only a single value without penalty. This construction is a better representation of biological systems than those that force the system to converge to a single value for objectives such as species concentrations, because it captures the intrinsic variability of these systems and it results in a large population of equally feasible parameter sets. A global solver that uses a scatter-search method followed by refinement with a local, gradient-based method handles the flat expanses of the search space. The sample code given in Eq. S2 demonstrates the implementation of this type of multi-objective, fuzzy cost function.

Necessarily, the solution in this case is not unique. Therefore, a set of 2500 optimizations was performed in which the model was run to steady-state, then evaluated against these objectives to generate a set of equally valid parameter vectors with which to initialize the model (Dataset S1); qualitatively, the number of optimizations does not affect the results. For this stage, the only species for which an initial condition was needed was microtubules; we assume 15 µM tubulin is present in abundance and excess over tau, and therefore do not include synthesis and degradation reactions for them. A total of 31 parameters were estimated. The resulting set of parameter vectors represents a population of neurons that behave in a healthy fashion and provides a way of evaluating the range of possible responses the system can display.

### Sensitivity of the healthy neuron population

**Figure 2:**
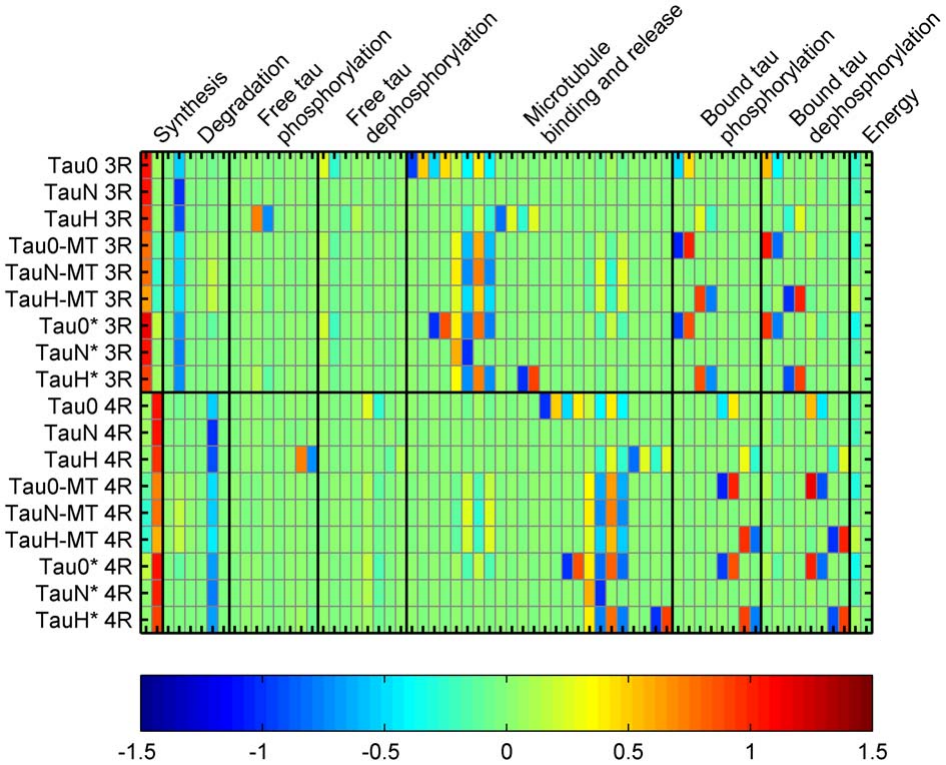
Relative, steady-state sensitivity for the healthy population of *in silico* neuron models. Median sensitivity coefficient at steady-state is shown for pairs of states (proteins) and parameters (rate constants). States and parameters associated with the chaperone and degradation are not shown, as this network is not engaged when the model is behaving in a manner consistent with a healthy neuron.

The median sensitivity of the population to perturbations in the parameters was calculated at steady-state, to provide insight into the triggers that disturb the system's homeostasis (Figure 2). The 95% confidence interval for the sensitivities was also calculated (Figure S4). Because the ratio of 3R to 4R tau is 1:1 in healthy neurons, the results for each are equivalent.

The identifiability of the sensitivity coefficients is defined by the span of the confidence interval; if the interval does not contain zero, the coefficient is considered identifiable. Although some small sensitivity coefficients are identifiable, most are not and the converse is true for larger coefficients, particularly those >0.5 (Figure S5). We find that changes in synthesis rates have the greatest positive impact on *in silico* homeostasis, while the perturbations in ubiquitin-independent degradation strongly and inversely alters the distribution of tau species. The situation for sensitivity to phosphorylation and dephosphorylation is more complex. Strong influences of this part of the network are found, but they do not act in concert. For example, aberrant 3R tau has a positive correlation with perturbations to the rate at with normal 3R tau is phosphorylated but it has an inverse relationship with the Michaelis-Menten constant. A similar situation is seen with bound tau states. The relationship between the microtubule interactions and tau distribution is similarly complex.

**Figure 3:**
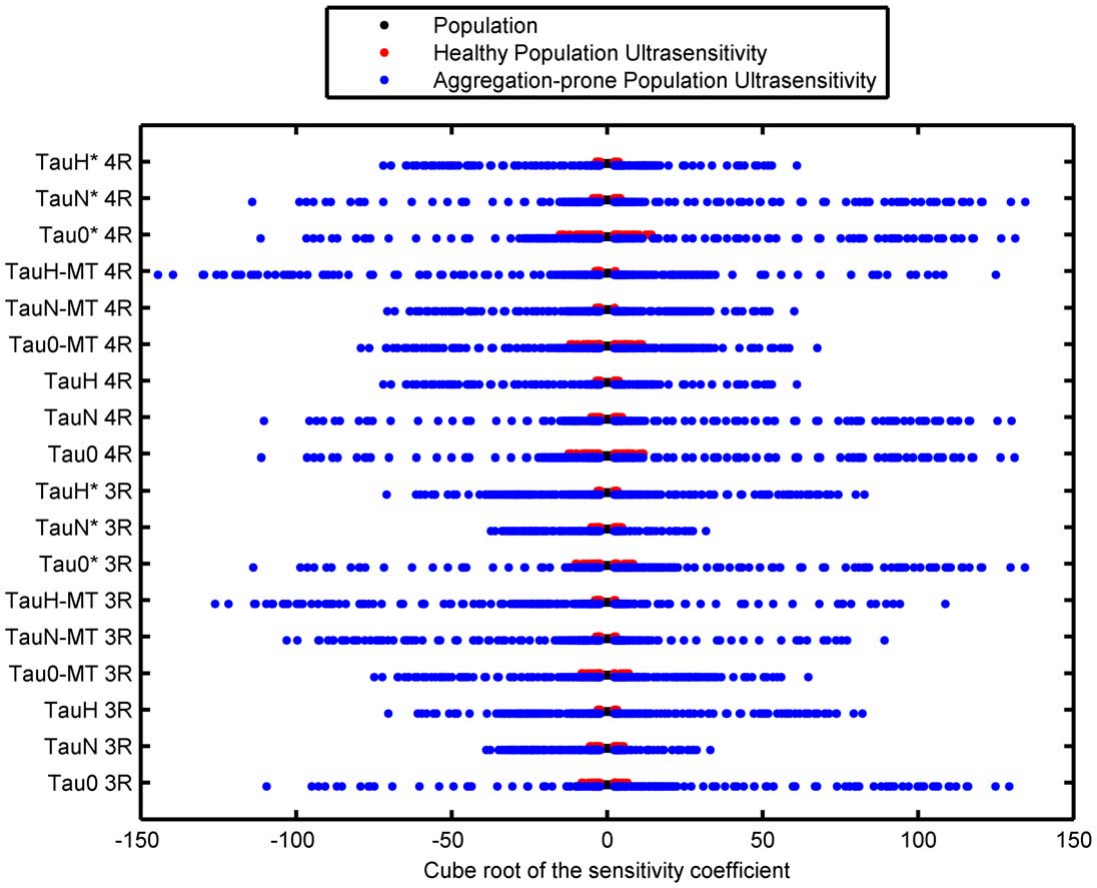
Ultrasensitivity in the populations. The cube root of the median sensitivity coefficients for each state across all parameters is shown with coefficients exceeding a 10-fold change in a state compared to the parameter perturbation highlighted. The ultrasensitive coefficients, which are denoted by the red and blue circled markers in the healthy and aggregation-prone populations, represent multiple individuals.

In Figure 3, the distribution of sensitivity coefficients within the healthy population is shown. The coefficients for each state were consolidated and transformed by the cube root, to accommodate the large scale and preserve the sign information of the coefficients. For all states, >99.9% of the coefficients fall below a value of 10, but in a few important cases high sensitivity to perturbations is observed. These individuals are relatively more vulnerable and less robust than the bulk of the population.

### Establishing aggregation-prone neuron models

For each model of a healthy neuron, we established a corresponding aggregation-prone model. The two models are coupled through the microtubule binding and release parameters. Synthesis, degradation, and phosphorylation and dephosphorylation were re-estimated because these activities are known to be altered in neurons containing tau aggregates. In addition, parameters associated with the chaperone and degradation machinery were estimated.

The objective function that quantifies the behavior of an aggregation-prone neuron is based on the data from several experiments. Quantification of tau in adult human brains affected by Alzheimer's was compared to that in control and showed that normal tau concentration was unaltered, but total tau concentration was 4–8 times normal tau; the increase is in the form of aberrant tau [40]. The critical concentration for aggregation is reported to be 0.2 µM [34]; necessarily, ubiquitinated tau approaches this concentration in an aggregation-prone neuron. The results of two silencing experiments were used to finalize the construction of the cost function corresponding to the aggregation-prone population [43]. In these experiments, silencing RNA was used to reduce the levels of Hsp70 and Hsp90 in COS-1 cells over-expressing human tau and the resulting effect on cytosolic (unbound) and microtubule-bound tau was assessed. A 50% reduction in Hsp70 resulted in a 5% decrease in unbound tau and a 75% decrease in bound tau, while a 75% reduction in Hsp90 resulted in a 10% decrease in unbound tau and a 70% decrease in bound tau [43]. The objective function was constructed as previously, resulting in the minimization of a function that is the sum of squared percent differences. For the “fuzzy” objectives, no cost was assigned if the model simulated a result in the allowable range of values.

Each result from the tuning of a neuron to healthy behavior was used to seed an optimization run designed to generate aggregation-prone behavior. For each run, the model was initialized to the steady-state concentrations achieved by the corresponding model of a healthy neuron. The simulation was run until quasi-steady-state was achieved and evaluated against the objective function to find parameters that instantiate an aggregation-prone model (Text S1).

**Figure 4:**
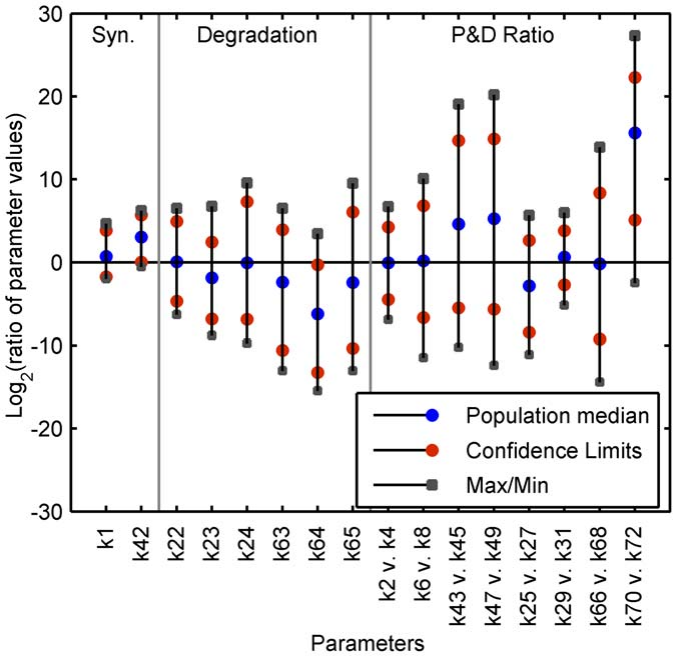
Parameter ratios. For parameters allowed to vary, the log_2_ ratios of the values in the aggregation-prone *vs.* the healthy population are given. The confidence intervals, maximum, and minimum for the population are also plotted.

In general, a single primary route to establish the aggregation-prone behavior was not obvious. Rather, the nature of the changes required to establish aggregation-prone neurons was multifactorial, although definite trends were observed in a small subset of the parameters (Figure 4). Confidence intervals (95%) were calculated and show just three identifiable trends; synthesis of 4R tau is generally increased while chaperone-independent degradation of normal 4R tau decreased, and the relative rate at which microtubule-bound, normal 4R tau was phosphorylated was elevated. Relative rate is a more meaningful measure of the change in phosphorylation and dephosphorylation processes and thus the metric on which we focus. The consistency with which these effects were observed suggests such behavior is likely to play a key role in initiating the pathological changes seen *in vivo*. As this result is consistent with the known increase in tau levels and decrease in proteasomal activity, and increased kinase that occurs in affected neurons, it provides a measure of validation for the model and encourages efforts to test the subsequent conclusions drawn from its behavior. In all cases, multiple perturbations in the rates of synthesis, degradation, and phosphorylation and dephosphorylation were required to induce an aggregation prone state.

### Sensitivity of the aggregation-prone population

The median sensitivity of the aggregation-prone population was calculated and the 95% confidence interval of the coefficients was used to determine their identifiability (Figures S6, S7, S8). As in the healthy population, synthesis and degradation are important processes with respect to tau distribution. Microtubule binding and phosphorylation and dephosphorylation are relatively less important in this population, although particularly for 3R tau a number of reactions in these processes are sensitive to tau distribution. Chaperone system reactions, on the other hand, do affect the behavior of the aggregation-prone population. Interestingly, the sensitivity to the aggregation reactions is only evident for aggregates; if the toxic moiety is actually soluble, aberrant tau, as it is increasingly thought, and not the aggregates then this has important ramifications for the selection of drug targets as the aggregation reactions have little effect on soluble tau.

**Figure 5:**
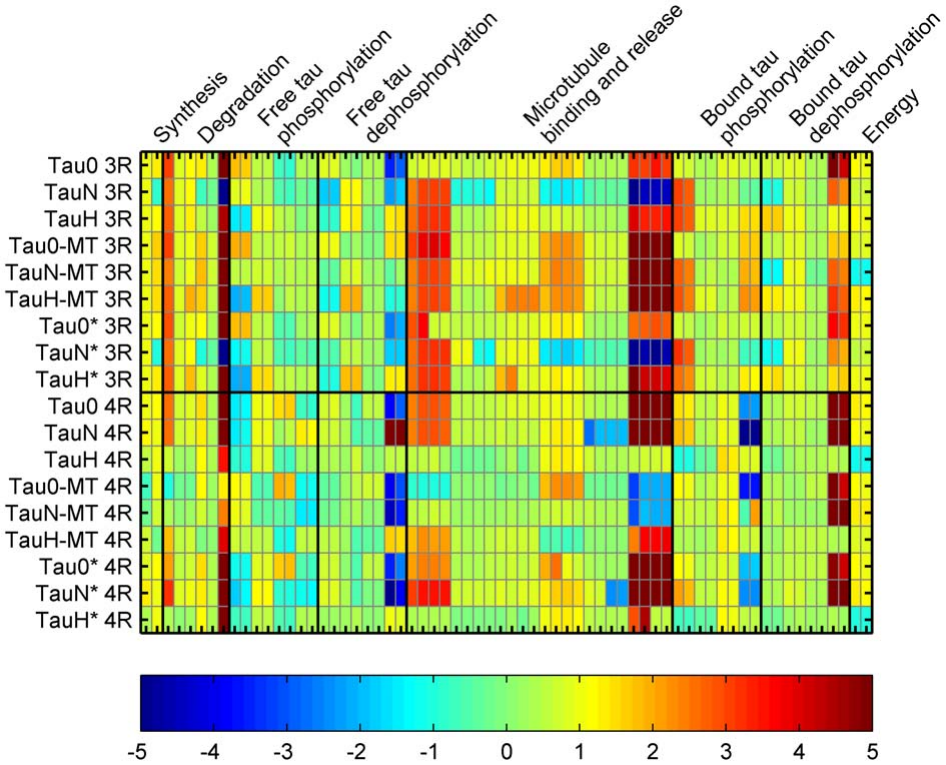
Sensitivity ratios. The ratio of the relative, median sensitivity coefficient for the aggregation prone population to the healthy population is shown for each state (protein concentration) and parameter (rate constant) pair.

To compare the aggregation-prone and healthy populations, the ratios between the sensitivity coefficients in each pair of matched individuals was calculated and the medians are shown in Figure 5. The aggregation-prone population exceeds twice the sensitivity of the healthy population 26% of the time and the magnitude of 46% of the median coefficients it is 2 fold lower. Notably, in nearly 24% of cases, the sign of the median sensitivity coefficient changes. This sign change is a striking and important phenomenon, as it suggests that the fundamental nature of the system's behavior changes during the transition from a healthy to an aggregation-prone state. It also suggests that the effect of changing conditions in the cell, due to drug treatment, for instance, depends on the state of the system. For example, the sensitivity of free and bound abnormal 4R tau species to phosphorylation shows a sign change; therefore, the efficacy of a treatment designed to influence phosphorylation reactions may depend upon the state of the system when treatment is initiated.

Evaluation of the distribution of the coefficients revealed subset of individuals with very large magnitude sensitivities to changing parameters, or ultrasensitivity (Figure 3). As with the healthy population, >99% of individuals were more moderately impacted by parameter perturbations, but the ultrasensitive individuals were of a much higher magnitude in this population. Additionally, this feature of ultrasensitivity was sharp and occurred after the accumulation of tau aggregates began. Systems such as this represent large obstacles to treatment; although sensitivity is required of a suitable drug target, the complex nature of the system's behavior in combination with ultrasensitivity is a challenging control problem and will make it difficult to re-establish homeostasis in these individuals. Disease progression independent of treatment is also significantly impacted by ultrasensitivity; cognitive decline is likely to be faster due to the fragile nature of this kind of network.

## Discussion

The *in silico* model developed to describe tau pathophysiology displays the very features of robustness and fragility that exist in real biological systems and these concepts are key to our understanding of the tauopathies. Indeed, the concept of robustness provides a framework in which disease can be understood as the inevitable consequence of a breakdown in the systems that normally maintain functionality [44]. Because these systems are complex, highly coupled, and nonlinear, their behavior is difficult to predict and systems-level approaches are required to understand and treat disease [45].

**Figure 6:**
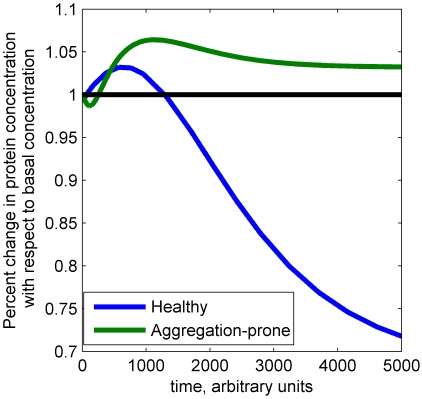
Time-dependent response to perturbations. A 5-fold increase in the parameter associated with the binding of normally phosphorylated, 3R tau to microtubules was applied and the response of unphosphorylated, microtubule bound 4R tau was monitored with respect to the basal concentration of this species. The perturbation results in a decrease in concentration with respect to basal in the healthy neuron, while in the aggregation-prone neuron, an increase in protein concentration is observed.

The population of healthy neurons is considered to be robust in several ways. The model generates healthy behavior in a relatively large domain of parameter space, a necessary property to maintain a phenotype given the inherent variation and noise in all biological systems. Likewise, the healthy population is robust and demands a vectorial assault to become pathological, as a multitude of perturbations to synthesis, degradation, and phosphorylation and dephosphorylation are required to generate a corresponding population of aggregation-prone neurons. In contrast, the aggregation-prone population is generally more sensitive to perturbations than the healthy population, as might be expected for a pathological phenotype (Figure 3). Moreover, the change in sign of a quarter of the sensitivity coefficients suggests that the fundamental behavior of this nonlinear system changes during the transition from healthy to aggregation-prone conditions. This change has implications for the drug discovery process; targeting such parts of the network is likely to be ineffective unless the timing is carefully considered. The case study shown in Figure 6 illustrates this point. In this individual, the binding of normally phosphorylated, 3R tau to microtubules was perturbed 5-fold and the concentration of microtubule bound, unphosphorylated 4R tau monitored in both the healthy and aggregation-prone states; the parameter perturbation is an *in silico* means of simulating drug treatment. Not only is an inverse response observed in each condition, but the qualitative response of the healthy neuron is in direct opposition to that of the aggregation-prone neuron. As the healthy and aggregation-prone neurons circumscribe the range of behaviors expected as a tauopathy advances, it logically follows that the sensitivity of relevant proteins to parameter perturbations switches at some point during disease progression. Such phenomenon may play an important role in the effectiveness of any particular drug, whose impact may be exactly the opposite of that intended and indeed even validated in experimental models. Therefore the identification of potential drug targets could be guided both by the identification of the perturbations that contribute to generate the diseased state and by the analysis of the parameter sensitivities in the healthy and diseased states. To minimize undesirable system-dependent effects, we suggest to target parameters for which the sign of the sensitivity coefficients does not change between the healthy and aggregation-prone states. Having identified synthesis, degradation, phosphorylation/dephosphorylation as keys to disease progression, the sensitivity coefficient associated synthesis and degradation reactions appeared to have a minimal number of changes of sign compared to the ones of phosphorylation/dephosphorylation reactions (Figure 5). From that point of view, synthesis and degradation appear to be preferential drug targets within the tau network.

A subset of the aggregation-prone population displays extreme fragility (Figure 3). This ultrasensitivity arises in the models of aggregation-prone neurons, and thus has implications for disease progression; the typical delay in diagnosing neurodegenerative diseases makes this phenomena potentially important with respect to treatment. While it is important to develop drugs that target sensitive points in biological networks, the widespread ultrasensitivity and nonlinearity observed in a subset of the population are likely to make the response of these systems difficult to predict or control, and they are likely to be highly resistant to rescue.

The robustness of the tau network and the multifactorial nature of its vulnerability to pathological change presents a challenge to the selection of drug targets, and for a subset of patients the disease is likely to be nearly impossible to reverse after the network becomes ultrasensitive. The model analysis also suggests that stalling or reversing tau pathophysiology will be further complicated by the timing at which the intervention is begun; a treatment may have an opposite effect on the system than is expected due to the sign inversion observed for some sensitivity coefficients.

The systems biology approach we have taken here has highlighted the complex, nonlinear behavior that cellular networks can display and suggests the difficulties the pharmaceutical and biotechnology industries will face in attempting to treat diseases associated with their aberrant functioning. By modeling both the physiological and the pathological functioning of the network governing tau function, we have shown that the biological response to a perturbation is dependent on the condition of the network and that, therefore, the time at which a compensatory perturbation is made is potentially significant. This implication is particularly relevant in therapeutic treatment timing and approach. The population-based analyses we have completed also highlights the importance of variability in the study and treatment of disease and the need to characterize the variability of the network components, such as reaction rates, to more fully elucidate its nature. Such variations are distinct from stochastic variation and the extent of the variability is likely dependent on the biological network and the particular network component. From a modeling perspective, *in silico* populations can be created for any model in a straightforward manner, by retaining not just a single optimization result but a number of results that fit the data almost equally well. As new experimental data is generated, the variation within the *in silico* populations will become more constrained and approach that seen *in vivo*. With respect to the optimization results, they suggest an approach that considers fitting matched measurements from the same individuals, for example if data was collected from individual animals over time, rather than taking a conglomerated value over measurements from multiple individuals. The computational, model-based approach to exploring cellular networks demonstrates a new paradigm for understanding disease that is likely to become increasingly effective as high-throughput and sequencing technologies quickly generate large databases of experimental data from which progressively more detailed, accurate models can be built.

## Methods

Current knowledge about the molecular biology of tau protein was integrated into a deterministic, kinetic model that was realized as a set of 45 ordinary differential equations (ODE's) (Tables S1 and S2) and implemented in MATLAB (Mathworks, Cambridge, UK). For each species, a differential equation was constructed from the rate equations for all reactions in which the species is involved; the reactions were modeled with mass action and Michaelis-Menten type kinetics. For example, a representative equation for the time-evolution of unphosphorylated tau is given by Eq. S1 (Text S1), which describes the change in concentration of unphosphorylated tau due to its synthesis, degradation, a conformational change that precedes microtubule binding, the restoration of the original protein conformation, phosphorylation, and dephosphorylation.

To validate the model construction effort, we used the method of Jacquez and Greif [46] to evaluate the *a priori* identifiability of the model and extended it to develop a suitable substitute for structural identifiability, as direct methods for evaluating structural identifiability are not feasible for large, nonlinear models such as this. In the traditional approach to *a priori* identifiability analyses, an iterative process of estimation and identifiability analysis is employed, reducing the number of parameters in the model after each iteration until the model is entirely identifiable [47]. We used a pseudo-global extension of this approach to diminish the parameter dependence of the results. First, Sobol' Low Discrepancy Sequences were used to generate 1024 points in parameter space. For each point, an *in silico* experiment in which tau was allowed to equilibrate for 2 hours after being induced in a tau-free system was simulated. We assumed all states were measurable and measured at 30-minute intervals during the 2 hour experiment. In addition, the local parametric sensitivity of the system was evaluated. From these simulated data, the correlation matrix M_c_ that establishes *a priori* identifiability was calculated according to Eq. S3 (Text S1).

Identifiable systems have correlations strictly < |1|. Here, the average correlation matrix is used to ascertain the identifiability of the system. Because the parameter sets were randomly generated, the resulting systems do not necessarily display biologically relevant behavior; therefore, the optimization objective function was calculated at each point in parameter space and used as weighting factors in calculating the average correlation. Given the model structure we established and the bounds on the parameter ranges, we can conclude that the model is *a priori* identifiable, but the high correlations between some parameters suggest that they might be difficult to estimate and therefore one parameter in each pair with a correlation >|0.95| was removed from the optimization and fixed to its nominal value.

Using this framework, that reduces parametric dependence and assumes all states are experimentally measurable, *a priori* identifiability is an acceptable proxy for structural identifiability. However the converse is not true and no conclusions can be drawn from a non-*a priori* identifiable system, as different experiments could reveal that the system is indeed structurally identifiable.

The model parameters were numerically fit using a hybrid stochastic-deterministic global optimization method [48], [49] that is based on well-established scatter search methods and implemented as a set of MATLAB functions, which are freely available on the authors' website and require only a single function call in MATLAB to implement. In brief, the method iterates between a global scatter search and local refinement of the solution using traditional methods; in this case we used MATLAB's fmincon, which is gradient-based technique, to perform this refinement. Although some experimentally derived kinetic data was available, it originated from heterogeneous sources including *in vitro* and *in vivo* platforms and under different experimental conditions. Therefore, generous bounds were used to define and explore parameter space.

To assess the effect of parameter perturbations on the steady-state concentrations of protein in the healthy population and quasi-steady-state (due to the polymerization reaction) concentration in the aggregation-prone population, the local, relative sensitivity of this system, given by Eq. S4 (Text S1), was evaluated.

The relative sensitivity coefficient gives the dependence of the protein concentration, “x_i_”, on a parameter, “p_j_” and is normalized with respect to the parameter and state values to facilitate. The non-normalized coefficients are calculated by applying the chain rule to Eq. S4 (Text S1), which results in a set of ordinary differential equations that give all the sensitivity coefficients associated with this system (Eq. S5, Text S1) by simultaneous integration of these sensitivity ODE's and the model ODE's in MATLAB. The sensitivity coefficients at steady-state were collected into a matrix, S_x_, of size N_x_ (number of states) by N_p_ (number of parameters).

## Supporting Information

Table S1 List of states, differential equations governing the time evolution of the states, and initial conditions for each state.
(0.11 MB PDF)

Table S2 List of reactions and the rate equations for the model of tau pathophysiology.
(0.18 MB PDF)

Text S1 Supporting equations for the construction of the ODE's and objective function and for the calculation of the correlation matrices and sensitivity coefficients.
(0.03 MB PDF)

Figure S1 Major events in the tau processing network. Phosphorylated (P) tau reversibly binds microtubules. In degenerating neurons, tau becomes abnormally and hyper-phosphorylated, misfolds, and is taken up by the chaperone system. Hsc70 mediates a decision between rescue and degradation.
(0.10 MB TIF)

Figure S2 Pseudo-global identifiability for the first stage of optimization to generate a population of healthy neuron models. The matrix shows the correlation between all pairs of parameters estimated during the optimization. A correlation of 1 or -1 indicates a non-identifiable parameter. No parameters were non-identifiable, but parameters that were highly correlated, i.e. >0.95 (circled), were nonetheless removed to improve the efficiency of the optimization.
(1.20 MB TIF)

Figure S3 Pseudo-global identifiability for the second stage of optimization to generate a population of aggregation-prone neuron models. The matrix shows the correlation between all pairs of parameters estimated during the optimization. A correlation of 1 or -1 indicates a non-identifiable parameter. No parameters were non-identifiable, nor did any parameter pairs have correlations greater than 0.95.
(1.60 MB TIF)

Figure S4 Identifiability of the median sensitivity coefficients for the healthy population, as computed from the 95% confidence intervals.
(0.32 MB TIF)

Figure S5 Distribution of the median sensitivity coefficients, categorized by their identifiability.
(0.07 MB TIF)

Figure S6 Relative, steady-state sensitivity for the aggregation-prone population of in silico neuron models. Median sensitivity coefficient at steady-state is shown for pairs of states (proteins) and parameters (rate constants). The parameters are grouped according to type.
(0.80 MB TIF)

Figure S7 Identifiability of the sensitivity coefficients, as computed from the 95% confidence intervals. If the confidence interval spanned 0, the coefficient was labeled unidentifiable.
(0.74 MB TIF)

Figure S8 Distribution of the median sensitivity coefficients of the aggregation-prone population according to their identifiability and magnitude.
(0.08 MB TIF)

Dataset S1 Optimization results.
(8.36 MB XLSX)
