# Supplementary material for: Vulnerabilities in the Tau Network and the Role of Ultrasensitive Points in Tau Pathophysiology
Source: PLoS Comput Biol. 2010 Nov 11;6(11):e1000997. doi: 10.1371/journal.pcbi.1000997 (PMC2978700; doi:10.1371/journal.pcbi.1000997)
Supplement: Table S1 — List of states, differential equations governing the time evolution of the states, and initial conditions for each state. (0.11 MB PDF) [file pcbi.1000997.s001.pdf]

Table S1. List of states, differential equations governing the time evolution of the states, and initial conditions for each state.

#	State	Species	Description	Differential Equation Governing the Time Evolution of the State	$[C]_{0,\ healthy}$ (μM)	Source
**1**	x(1)	Tau03R	Newly synthesized 3R tau, unphosphorylated	dxdt(1)= r1-r2+r3-r6+r7-r18+r29;	0	*Results are independent of the initial condition for 3R tau.
**2**	x(2)	TauN3R	Normally phoshporylated 3R tau	dxdt(2)= r2-r3-r4+r5-r10+r11-r19;	0	
**3**	x(3)	TauH3R	Misfolded 3R tau prone to aggregation	dxdt(3)= r4-r5-r14+r15-r20-r25+r26;	0	
**4**	x(4)	Tau03RMT	Unphosphorylated 3R tau bound to microtubule	dxdt(4)= r8-r9-r21+r22;	0	
**5**	x(5)	TauN3RMT	Normal 3R tau bound to microtubule	dxdt(5)= r12-r13+r21-r22-r23+r24;	0	
**6**	x(6)	TauH3RMT	Misfolded 3R tau bound to microtubule	dxdt(6)= r16-r17+r23-r24;	0	
**7**	x(7)	Tau0*3R	Conformationally altered, unphosphorylated, 3R tau with affinity for MT	dxdt(7)= r6-r7-r8+r9;	0	
**8**	x(8)	TauN*3R	Conformationally altered, normal, 3R tau with affinity for MT	dxdt(8)= r10-r11-r12+r13;	0	
**9**	x(9)	TauH*3R	Conformationally altered, misfolded, 3R tau with affinity for MT	dxdt(9)= r14-r15-r16+r17;	0	
**10**	x(10)	MT	Microtubules	dxdt(10)= -r8+r9-r12+r13-r16+r17-r42+r43-r46+r47-r50+r51;	15	
**11**	x(11)	Hsc70	Chaperone	dxdt(11)= -r25+r26+r27+r31 - r59 + r60 + r61 + r65;	0.1	Assumed
**12**	x(12)	Hsp90	Refolding chaperone	dxdt(12)= -r27+r29 - r61+r63;	0.1	Assumed
**13**	x(13)	Bag2	Bag2	dxdt(13)= -r32 + r33 - r66 + r67;	0.1	Assumed
**14**	x(14)	CHIP	Degrading chaperone	dxdt(14)= -r30+r31+r33-r64+r65+r67;	0.1	Assumed
**15**	x(15)	TauH3R-Hsc70	Protein triage complex, 3R tau	dxdt(15)= r25-r26-r27-r30+r33;	0	
**16**	x(16)	TauH3R-Hsp90	Refolding complex, 3R tau	dxdt(16)= r27-r28;	0	
**17**	x(17)	Tau03R-Hsp90	Refolding complex with restored substrate, 3R tau	dxdt(17)= r28-r29;	0	
**18**	x(18)	TauH3R-CHIP-Hsc70	Degradation complex, 3R tau	dxdt(18)= r30-r31-r32;	0	
**19**	x(19)	TauH3R-CHIP-Hsc70-Bag2	Protein triage complex, 3R tau	dxdt(19)= r32-r33;	0	
20	x(20)	TauH3RUb	Ubiquitinated 3R tau	dxdt(20)= r31-r34 - 2*r71 + 2*r72 - r75 + r76 - r77 - r78 + r79;	0	
**21**	x(21)	20S	20S proteasome	dxdt(21)= 0;	1	Assumed
**22**	x(22)	26S	26S proteasome	dxdt(22)= 0;	1	Assumed
23	x(23)	ATP	Energy	dxdt(23)= (-1)*(r2+r4+r18+r19+r20+r21+r23+r34+r36+r38+r52+r53+r54+r55 + r57 + r68-r69+r7	0	
24	x(24)	ADP	Energy	dxdt(24)= r2+r4+r18+r19+r20+r21+r23+r34+r36+r38+r52+r53+r54+r55 + r57 + r68-r69+r70;	1	
**25**	x(25)	Tau04R	Newly synthesized 4R tau, unphosphorylated	dxdt(25)= r35-r40+r41-r36+r37-r52+r63;	0	*Results are independent of the initial condition for 4R tau.
**26**	x(26)	TauN4R	Normally phoshporylated 4R tau	dxdt(26)= r36-r37-r38+r39-r44+r45-r53;	0	
**27**	x(27)	TauH4R	Misfolded 4R tau prone to aggregation	dxdt(27)= r38-r39-r48+r49-r54-r59+r60;	0	
**28**	x(28)	Tau04MT	Unphosphorylated 4R tau bound to microtubule	dxdt(28)= r42-r43-r55+r56;	0	
**29**	x(29)	TauN4RMT	Normal 4R tau bound to microtubule	dxdt(29)= r46-r47+r55-r56-r57+r58;	0	
**30**	x(30)	TauH4RMT	Misfolded 4R tau bound to microtubule	dxdt(30)= r50-r51+r57-r58;	0	
**31**	x(31)	Tau0*4R	Conformationally altered, unphosphorylated, 4R tau with affinity for MT	dxdt(31)= r40-r41-r42+r43;	0	
**32**	x(32)	TauN*4R	Conformationally altered, normal, 4R tau with affinity for MT	dxdt(32)= r44-r45-r46+r47;	0	
**33**	x(33)	TauH*4R	Conformationally altered, misfolded, 4R tau with affinity for MT	dxdt(33)= r48-r49-r50+r51;	0	
**34**	x(34)	TauH4R-Hsc70	Protein triage complex, 4R tau	dxdt(34)= r59-r60-r61-r64+r67;	0	
**35**	x(35)	TauH4R-Hsp90	Refolding complex, 4R tau	dxdt(35)= r61-r62;	0	
**36**	x(36)	Tau04R-Hsp90	Refolding complex with restored substrate, 4R tau	dxdt(36)= r62-r63;	0	
**37**	x(37)	TauH4R-CHIP-Hsc70	Degradation complex, 4R tau	dxdt(37)= r64-r65-r66;	0	
**38**	x(38)	TauH4R-CHIP-Hsc70-Bag2	Protein triage complex, 4R tau	dxdt(38)= r66-r67;	0	
39	x(39)	TauH4RUB	Ubiquitinated 4R tau	dxdt(39)= r65-r68-2*r73+2*r74-r80+r81-r82-r83+r84;	0	
40	x(40)	Nucleus (3R tau)	Nucleus for aggregation (dimer) from 3R tau	dxdt(40) = r71-r72-r75+r76;	0	
41	x(41)	Nucleus (4R tau)	Nucleus for aggregation (dimer) from 4R tau	dxdt(41) = r73-r74-r80+r81;	0	
42	x(42)	Length 3 aggregate of 3R tau	Aggregates of length 3, 3R tau	dxdt(42) = r75-r76-r77;	0	
43	x(43)	3R aggregates > length 3 (Ap)	Aggregates greater than length 3, 3R tau	dxdt(43) = r77;	0	
44	x(44)	Length 3 aggregates of 4R tau	Aggregates of length 3, 4R tau	dxdt(44) = r80-r81-r82;	0	
45	x(45)	4R aggregates > length 3 (Bp)	Aggregates greater than length 3, 4R tau	dxdt(45) = r82;	0	
