# Supplementary material for: Vulnerabilities in the Tau Network and the Role of Ultrasensitive Points in Tau Pathophysiology
Source: PLoS Comput Biol. 2010 Nov 11;6(11):e1000997. doi: 10.1371/journal.pcbi.1000997 (PMC2978700; doi:10.1371/journal.pcbi.1000997)
Supplement: Table S2 — List of reactions and the rate equations for the model of tau pathophysiology. (0.18 MB PDF) [file pcbi.1000997.s002.pdf]

Table S2. List of reactions and the rate equations for the model of tau pathophysiology.

Reaction Type	Description	Kinetic Law	Reactants	Products	Params	Units
**Synthesis of 3R tau**	Tau synthesis	r(1) = k(1);	-	Tau0	k(1)	μM-s^{-1}
**Phosphorylation & dephosphorylation of free 3R tau**	Phosphorylation of newly synthesized tau	r(2) = k(2)*x(1)*x(23)/(k(3)+x(1));	Tau0, ATP	TauN, ADP	k(2)<br>k(3)	μM^{-1}-s^{-1}<br>μM
	Dephosphorylation of normally phosphorylated tau	r(3) = k(4)*x(2)/(k(5)+x(2));	TauN	Tau0	k(4)<br>k(5)	s^{-1}<br>μM
	Phosphorylation of normally phosphorylated tau	r(4) = k(6)*x(2)*x(23)/(k(7)+x(2));	TauN, ATP	TauH, ADP	k(6)<br>k(7)	μM^{-1}-s^{-1}<br>μM
	Dephosphorylation of abnormal/misfolded tau	r(5) = k(8)*x(3)/(k(9)+x(3));	TauH	TauN	k(8)<br>k(9)	s^{-1}<br>μM
**Microtubule binding and release of 3R tau species**	Conformation change favoring MT binding	r(6) = k(10)*x(1);	Tau0	Tau0*	k(10)	s^{-1}
	Conformational change back to original	r(7) = k(11)*x(7);	Tau0*	Tau0	k(11)	s^{-1}
	Binding of newly synthesized tau to MT	r(8) = k(12)*x(7)*x(10);	Tau0*, MT	Tau0*-MT	k(12)	μM^{-1}-s^{-1}
	Release of newly synthesized tau from MT	r(9) = k(13)*x(4);	Tau0*-MT	Tau0*, MT	k(13)	s^{-1}
	Conformation change favoring MT binding	r(10) = k(14)*x(2);	TauN	TauN*	k(14)	s^{-1}
	Conformational change back to original	r(11) = k(15)*x(8);	TauN*	TauN	k(15)	s^{-1}
	Binding of normally phosphorylated tau to MT	r(12) = k(16)*x(8)*x(10);	TauN*, MT	TauN*-MT	k(16)	μM^{-1}-s^{-1}
	Release of normally phosphorylated tau from MT	r(13) = k(17)*x(5);	TauN*-MT	TauN*, MT	k(17)	s^{-1}
	Conformation change favoring MT binding	r(14) = k(18)*x(3);	TauH	TauH*	k(18)	s^{-1}
	Conformational change back to original	r(15) = k(19)*x(9);	TauH*	TauH	k(19)	s^{-1}
	Binding of misfolded tau to MT	r(16) = k(20)*x(9)*x(10);	TauH*, MT	TauH*-MT	k(20)	μM^{-1}-s^{-1}
	Release of misfolded tau from microtubules	r(17) = k(21)*x(6);	TauH*-MT	TauH*, MT	k(21)	s^{-1}
**Free 3R tau degradation by non-ubiquitin dependent proteasome**	Degradation of newly synthesized tau	r(18) = k(22)*x(1)*x(21)*x(23);	Tau0, 20S, ATP	ADP, 20S	k(22)	μM^{-2}-s^{-1}
	Degradation of phosphorylated tau	r(19) = k(23)*x(2)*x(21)*x(23);	TauN, 20S, ATP	ADP, 20S	k(23)	μM^{-2}-s^{-1}
	Degradation of misfolded tau	r(20) = k(24)*x(3)*x(21)*x(23);	TauH, 20S, ATP	ADP, 20S	k(24)	μM^{-2}-s^{-1}
**Phosphorylation & dephosphorylation of microtubule-bound 3R tau**	Phosphorylation of MT-bound newly synthesized tau	r(21) = k(25)*x(4)*x(23)/(k(26)+x(4));	Tau0*-MT, ATP	TauN*-MT	k(25)<br>k(26)	μM^{-1}-s^{-1}<br>μM
	Dephosphorylation of MT-bound normally phosphorylated tau	r(22) = k(27)*x(5)/(k(28)+x(5));	TauN*-MT	Tau0*-MT	k(27)<br>k(28)	s^{-1}<br>μM
	Phosphorylation of MT-bound normally phosphorylated tau	r(23) = k(29)*x(5)*x(23)/(k(30)+x(5));	TauN*-MT, ATP	TauH-MT	k(29)<br>k(30)	μM^{-1}-s^{-1}<br>μM
	Dephosphorylation of MT-bound abnormal/misfolded tau	r(24) = k(31)*x(6)/(k(32)+x(6));	TauH-MT	TauN*-MT	k(31)<br>k(32)	s^{-1}<br>μM
**3R Tau loading by Hsp70**	Association of misfolded tau with Hsp70	r(25) = k(33)*x(3)*x(11);	TauH, Hsp70	TauH-Hsp70	k(33)	μM^{-1}-s^{-1}
	Hsp70 release	r(26) = k(34)*x(15);	TauH-Hsp70	TauH, Hsp70	k(34)	s^{-1}
**3R Tau Refolding**	Exchange of Hsp70 for Hsp90	r(27) = k(35)*x(15)*x(12);	TauH-Hsc70, Hsp90	TauH-Hsp90, Hsc70	k(35)	μM^{-1}-s^{-1}
	Restoration of TauH to Tau0 via Hsp90	r(28) = k(8)*x(16)/(k(9)+x(16));	TauH-Hsc90	Tau0-Hsp90	k(8)<br>k(9)	s^{-1}<br>μM
	Release of Tau0 from Hsp90	r(29) = k(36)*x(17);	Tau0-Hsp90	Hsp90, Tau0	k(36)	s^{-1}
**Degradation of 3R tau by chaperones**	Exchange of Hsc70 for CHIP	r(30) = k(37)*x(15)*x(14);	TauH-Hsc70, CHIP	TauH-Hsc70-CHIP	k(37)	μM^{-1}-s^{-1}
	Ubiquitination of tau	r(31) = k(38)*x(18);	TauH-Hsc70-CHIP	TauHUb, Hsc70, CHIP	k(38)	s^{-1}
	Binding of Bag-2 to the degradation complex	r(32) = k(39)*x(18)*x(13);	TauH-Hsc70-CHIP, Bag2	TauH-Hsc70-CHIP-Bag2	k(39)	μM^{-1}-s^{-1}
	Rescue from degradation by Bag2	r(33) = k(40)*x(19);	TauH-Hsc70-CHIP-Bag2	TauH-Hsc70, CHIP, Bag2	k(40)	s^{-1}
	Degradation of ubiquitinated tau	r(34) = k(41)*x(20)*x(22)*x(23);	TauHUb, 26S, ATP	ADP, 26S	k(41)	μM^{-2}-s^{-1}
**Synthesis of 4R tau**	Synthesis of 4R tau	r(35) = k(42);	-	Tau0	k(42)	μM-s^{-1}
**Phosphorylation & dephosphorylation of free 4R tau**	Phosphorylation of newly synthesized tau	r(36) = k(43)*x(25)*x(23)/(k(44)+x(25));	Tau0, ATP	TauN, ADP	k(43)<br>k(44)	μM^{-1}-s^{-1}<br>μM
	Dephosphorylation of normally phosphorylated tau	r(37) = k(45)*x(26)/(k(46)+x(26));	TauN	Tau0	k(45)<br>k(46)	s-1<br>μM
	Phosphorylation of normally phosphorylated tau	r(38) = k(47)*x(26)*x(23)/(k(48)+x(26));	TauN, ATP	TauH, ADP	k(47)<br>k(48)	μM^{-1}-s^{-1}<br>μM
	Dephosphorylation of abnormal/misfolded tau	r(39) = k(49)*x(27)/(k(50)+x(27));	TauH	TauN	k(49)<br>k(50)	s^{-1}<br>μM
**Microtubule binding and release of 4R tau species**	Conformation change favoring MT binding	r(40) = k(51)*x(25);	Tau0	Tau0*	k(51)	s^{-1}
	Conformational change back to original	r(41) = k(52)*x(31);	Tau0*	Tau0	k(52)	s^{-1}
	Binding of newly synthesized tau to MT	r(42) = k(53)*x(31)*x(10);	Tau0*, MT	Tau0*-MT	k(53)	μM^{-1}-s^{-1}
	Release of newly synthesized tau from MT	r(43) = k(54)*x(28);	Tau0*-MT	Tau0*, MT	k(54)	s^{-1}
	Conformation change favoring MT binding	r(44) = k(55)*x(26);	TauN	TauN*	k(55)	s^{-1}
	Conformational change back to original	r(45) = k(56)*x(32);	TauN*	TauN	k(56)	s^{-1}
	Binding of normally phosphorylated tau to MT	r(46) = k(57)*x(32)*x(10);	TauN*, MT	TauN*-MT	k(57)	μM^{-1}-s^{-1}
	Release of normally phosphorylated tau from MT	r(47) = k(58)*x(29);	TauN*-MT	TauN*, MT	k(58)	s^{-1}
	Conformation change favoring MT binding	r(48) = k(59)*x(27);	TauH	TauH*	k(59)	s^{-1}
	Conformational change back to original	r(49) = k(60)*x(33);	TauH*	TauH	k(60)	s^{-1}
	Binding of misfolded tau to MT	r(50) = k(61)*x(33)*x(10);	TauH*, MT	TauH*-MT	k(61)	μM^{-1}-s^{-1}
	Release of misfolded tau from microtubules	r(51) = k(62)*x(30);	TauH*-MT	TauH*, MT	k(62)	s^{-1}
**Free 4R tau degradation by non-ubiquitin dependent proteasome**	Degradation of newly synthesized tau	r(52) = k(63)*x(25)*x(23)*x(21);	Tau0, 20S, ATP	ADP, 20S	k(63)	μM^{-2}-s^{-1}
	Degradation of phosphorylated tau	r(53) = k(64)*x(26)*x(23)*x(21);	TauN, 20S, ATP	ADP, 20S	k(64)	μM^{-2}-s^{-1}
	Degradation of misfolded tau	r(54) = k(65)*x(27)*x(23)*x(21);	TauH, 20S, ATP	ADP, 20S	k(65)	μM^{-2}-s^{-1}
**Phosphorylation & dephosphorylation of microtubule-bound 4R tau**	Phosphorylation of MT-bound newly synthesized tau	r(55) = k(66)*x(28)*x(23)/(k(67)+x(28));	Tau0*-MT, ATP	TauN*-MT	k(66)<br>k(67)	μM^{-1}-s^{-1}<br>μM
	Dephosphorylation of MT-bound normally phosphorylated tau	r(56) = k(68)*x(29)/(k(69)+x(29));	TauN*-MT	Tau0*-MT	k(68)<br>k(69)	s^{-1}<br>μM
	Phosphorylation of MT-bound normally phosphorylated tau	r(57) = k(70)*x(29)*x(23)/(k(71)+x(29));	TauN*-MT, ATP	TauH-MT	k(70)<br>k(71)	μM^{-1}-s^{-1}<br>μM
	Dephosphorylation of MT-bound abnormal/misfolded tau	r(58) = k(72)*x(30)/(k(73)+x(30));	TauH-MT	TauN*-MT	k(72)<br>k(73)	s^{-1}<br>μM
**4R Tau loading by Hsp70**	Association of misfolded tau with Hsp70	r(59) = k(74)*x(27)*x(11);	TauH, Hsp70	TauH-Hsp70	k(74)	μM^{-1}-s^{-1}
	Hsp70 release	r(60) = k(75)*x(34);	TauH-Hsp70	TauH, Hsp70	k(75)	s^{-1}
**4R Tau Refolding**	Exchange of Hsp70 for Hsp90	r(61) = k(76)*x(34)*x(12);	TauH-Hsc70, Hsp90	TauH-Hsp90, Hsc70	k(76)	μM^{-1}-s^{-1}
	Restoration of TauH to Tau0 via Hsp90	r(62) = k(77)*x(35)/(k(9)+x(35));	TauH-Hsc90	Tau0-Hsp90	k(77) = k(8)<br>k(9)	s^{-1}<br>μM
	Release of Tau0 from Hsp90	r(63) = k(78)*x(36);	Tau0-Hsp90	Hsp90, Tau0	k(78)	s^{-1}
**Degradation of 4R tau by chaperones**	Exchange of Hsc70 for CHIP	r(64) = k(79)*x(34)*x(14);	TauH-Hsc70, CHIP	TauH-Hsc70-CHIP	k(79)	μM^{-1}-s^{-1}
	Ubiquitination of tau	r(65) = k(80)*x(37);	TauH-Hsc70-CHIP	TauHUb, Hsc70, CHIP	k(80)	s^{-1}
	Binding of Bag-2 to the degradation complex	r(66) = k(81)*x(37)*x(13);	TauH-Hsc70-CHIP, Bag2	TauH-Hsc70-CHIP-Bag2	k(81)	μM^{-1}-s^{-1}
	Rescue from degradation by Bag2	r(67) = k(82)*x(38);	TauH-Hsc70-CHIP-Bag2	TauH-Hsc70, CHIP, Bag2	k(82)	s^{-1}
	Degradation of ubiquitinated tau	r(68) = k(83)*x(39)*x(22)*x(23);	TauHUb, 26S, ATP	ADP, 26S	k(83)	μM^{-2}-s^{-1}
**ATP generation and use by other processes**	Generation of ATP	r(69) = k(84)*x(24);	ADP	ATP	k(84)	s^{-1}
	Use of ATP by other processes	r(70) = k(85)*x(23);	ATP	ADP	k(85)	s^{-1}
**Aggregation of 3R and 4R tau**	Nucleation of Tau3	r(71) = k(86)*x(20).^2;	TauH3RUb (2)	Nucleus3	k(86)	μM^{-1}-s^{-1}
	Dissociation of Tau3 Nucleus	r(72) = k(87)*x(40);	Nucleus3	TauH3RUb (2)	k(87)	s^{-1}
	Nucleation of Tau4	r(73) = k(88)*x(39).^2;	TauH4RUb (2)	Nucleus4	k(88)	μM^{-1}-s^{-1}
	Dissociation of Tau4 Nucleus	r(74) = k(89)*x(41);	Nucleus4	TauH4RUb (2)	k(89)	s^{-1}
	Formation of Tau3, length 3	r(75) = k(90)*x(20)*x(40);	Nucleus3, TauH3RUB	Agg33	k(90)	μM^{-1}-s^{-1}
	Dissociation	r(76) = k(91)*x(42);	Agg33	Nucleus3, TauH3RUB	k(91)	s^{-1}
	Formation of Tau3, l > 3 from Tau3 aggregate of length 3	r(77) = k(90)*x(20)*x(42);	TauH3RUB, Agg33	Ap	k(90)	μM^{-1}-s^{-1}
	Formation of agg of l > 3 from aggregates of l > 3	r(78) = k(90)*x(20)*x(43);	TauH3RUB, Ap	Ap	k(90)	μM^{-1}-s^{-1}
	Dissociation of aggregates l>3	r(79) = k(91)*x(43);	Ap	TauH3RUB, Ap	k(91)	s^{-1}
	Formation of Tau4, length 3	r(80) = k(92)*x(39)*x(41);	Nucleus4, TauH4RUB	Agg43	k(92)	μM^{-1}-s^{-1}
	Dissociation	r(81) = k(93)*x(44);	Agg43	Nucleus4, TauH4RUB	k(93)	s^{-1}
	Formation of Tau4, l > 3 from Tau3 aggregate of length 3	r(82) = k(92)*x(39)*x(44);	TauH4RUb, Agg43	Bp	k(92)	μM^{-1}-s^{-1}
	Formation of agg of l > 3 from aggregates of l > 3	r(83) = k(92)*x(39)*x(45);	TauH4RUb,Bp	Bp	k(92)	μM^{-1}-s^{-1}
	Dissociation of aggregates l>3	r(84) = k(93)*x(45);	Bp	TauH4RUb, Bp	k(93)	s^{-1}
