# Supplementary material for: Vulnerabilities in the Tau Network and the Role of Ultrasensitive Points in Tau Pathophysiology
Source: PLoS Comput Biol. 2010 Nov 11;6(11):e1000997. doi: 10.1371/journal.pcbi.1000997 (PMC2978700; doi:10.1371/journal.pcbi.1000997)
Supplement: Text S1 — Supporting equations for the construction of the ODE's and objective function and for the calculation of the correlation matrices and sensitivity coefficients. (0.03 MB PDF) [file pcbi.1000997.s003.pdf]

Supporting Text

Supplementary Equations

Equation S1 is a representative ordinary differential equation (ODE) to describe the time-evolution of protein concentrations in the model. This particular equation describes the evolution of unphosphorylated tau due to its synthesis, degradation, a conformational change that precedes microtubule binding, the restoration of the original protein conformation, phosphorylation, and dephosphorylation. The (ODE's) for the remaining protein species were similarly assembled.

$$\frac{d[Tau]}{dt} = k_{synthesis} - k_{deg}[Tau] - k_{conf}[Tau] + k_{restore}[Tau] - \frac{k_{phos}[Tau][ATP]}{K_m + [Tau]} + \frac{k_{dephos}\left[Tau^P\right][ATP]}{K_m + [Tau]} \qquad (S1)$$

Equation S2 demonstrates the implementation of the multi-objective, fuzzy cost function used in the optimization of the healthy and aggregation-prone populations.

$$\begin{aligned}
&if\ x < 5 \\
&\quad obj_1 = \left(\frac{x-5}{5}\right)^2 \\
&elseif\ x > 10 \\
&\quad obj_1 = \left(\frac{x-10}{10}\right)^2 \\
&else \\
&\quad obj_1 = 0 \\
&end \\
&\vdots \\
&Total\cos t = \sum_i obj_i
\end{aligned} \qquad (S2)$$

Equation S3 shows the construction of the correlation matrix, M_c, that establishes *a priori* identifiability. S_y is an N_x (number of measurable states) by N_p (number of parameters) sensitivity matrix.

$$M_c = \left(\begin{bmatrix} S_y(t_1) \\ S_y(t_2) \\ \vdots \\ S_y(t_n) \end{bmatrix}\right) \qquad (S3)$$

Equation S4 gives the mathematical definition of a sensitivity coefficient as the change in state, x_i, with respect to a change in parameter, p_j, at time t_k. The coefficients are normalized to the state and parameter values to facilitate comparisons among the coefficients for different states and parameters, whose magnitude may vary widely.

$$s_{ij}(t_k) = \frac{\partial x_i(t_k)}{\partial p_j} * \frac{p_j}{x_i(t_k)} \qquad \text{(S4)}$$

The non-normalized sensitivity coefficients are calculated by applying the chain rule to Eq. S4, which results in the set of ordinary differential equations given in Equation S5. The simultaneous integration of the sensitivity ODE's and the model ODE's gives the sensitivity for every state/parameter pair.

$$\dot{s}_{ij} = \frac{\partial f}{\partial x_i} s_{ij} + \frac{\partial f}{\partial p_j} \qquad \text{(S5)}$$
