# Supplementary figures and images for: Vulnerabilities in the Tau Network and the Role of Ultrasensitive Points in Tau Pathophysiology
Source: PLoS Comput Biol. 2010 Nov 11;6(11):e1000997. doi: 10.1371/journal.pcbi.1000997 (PMC2978700; doi:10.1371/journal.pcbi.1000997)

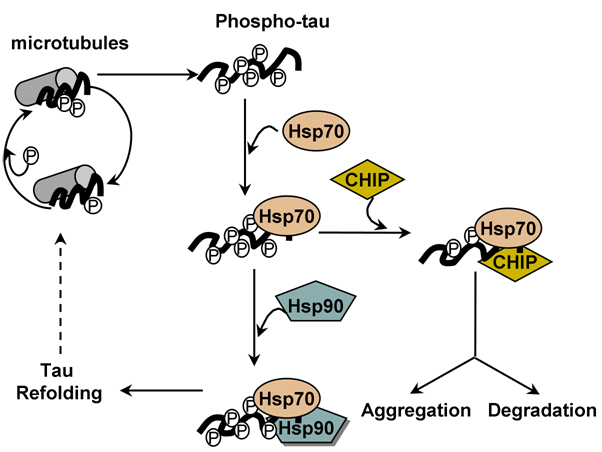

Supplement: Figure S1 — Major events in the tau processing network. Phosphorylated (P) tau reversibly binds microtubules. In degenerating neurons, tau becomes abnormally and hyper-phosphorylated, misfolds, and is taken up by the chaperone system. Hsc70 mediates a decision between rescue and degradation. (0.10 MB TIF) [file pcbi.1000997.s004.tif]

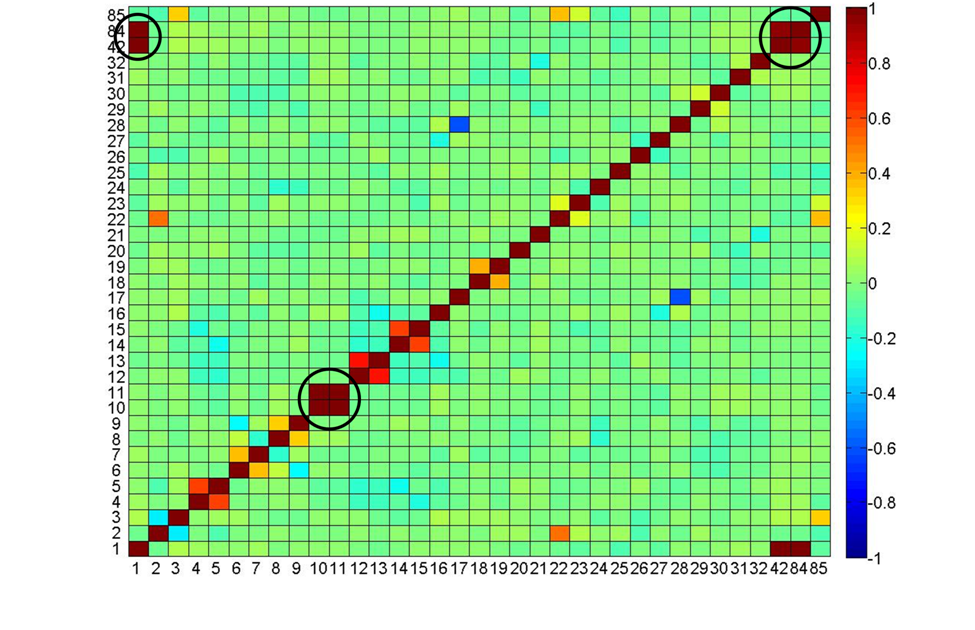

Supplement: Figure S2 — Pseudo-global identifiability for the first stage of optimization to generate a population of healthy neuron models. The matrix shows the correlation between all pairs of parameters estimated during the optimization. A correlation of 1 or -1 indicates a non-identifiable parameter. No parameters were non-identifiable, but parameters that were highly correlated, i.e. >0.95 (circled), were nonetheless removed to improve the efficiency of the optimization. (1.20 MB TIF) [file pcbi.1000997.s005.tif]

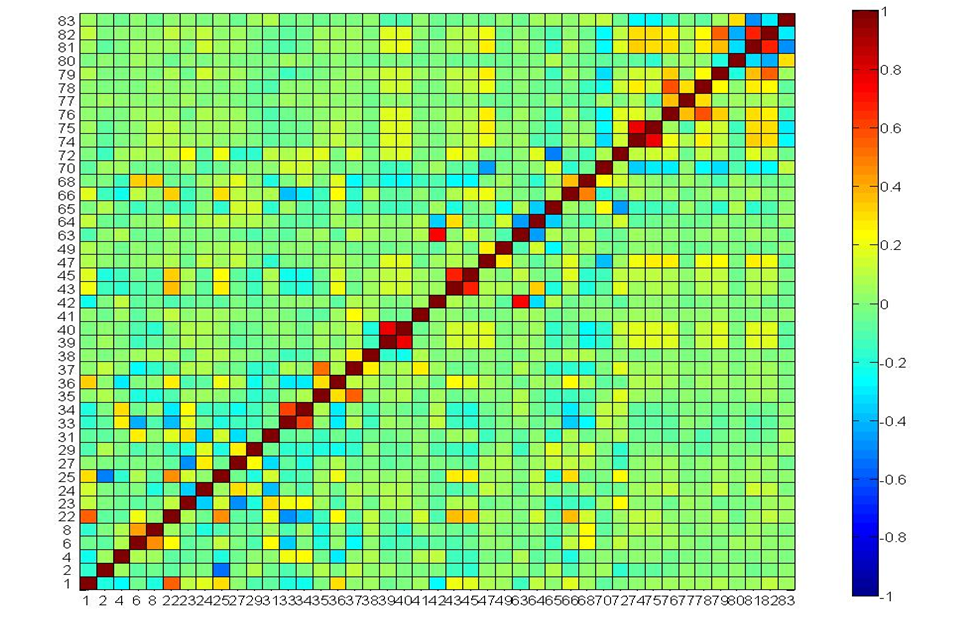

Supplement: Figure S3 — Pseudo-global identifiability for the second stage of optimization to generate a population of aggregation-prone neuron models. The matrix shows the correlation between all pairs of parameters estimated during the optimization. A correlation of 1 or -1 indicates a non-identifiable parameter. No parameters were non-identifiable, nor did any parameter pairs have correlations greater than 0.95. (1.60 MB TIF) [file pcbi.1000997.s006.tif]

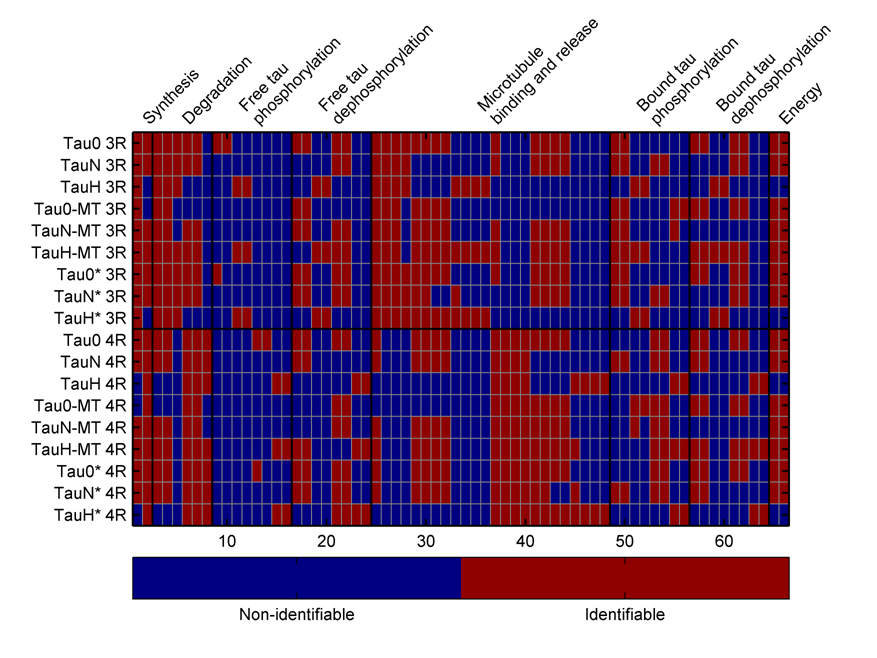

Supplement: Figure S4 — Identifiability of the median sensitivity coefficients for the healthy population, as computed from the 95% confidence intervals. (0.32 MB TIF) [file pcbi.1000997.s007.tif]

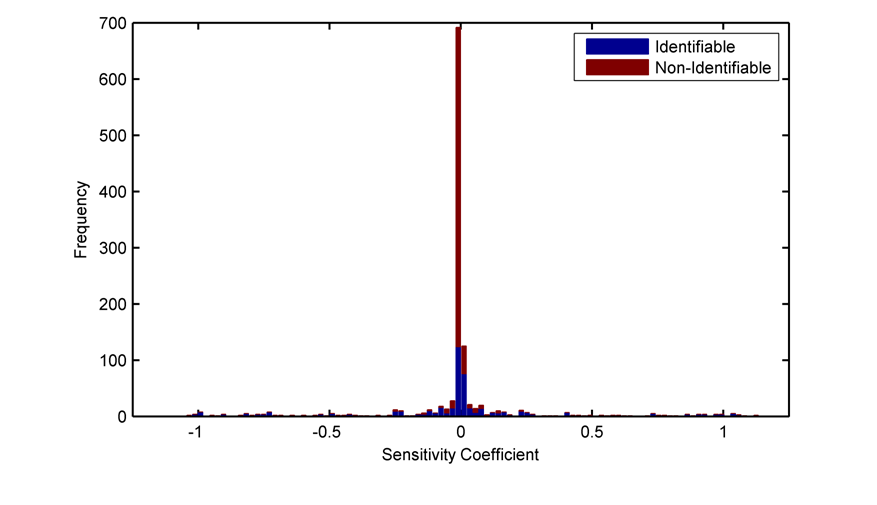

Supplement: Figure S5 — Distribution of the median sensitivity coefficients, categorized by their identifiability. (0.07 MB TIF) [file pcbi.1000997.s008.tif]

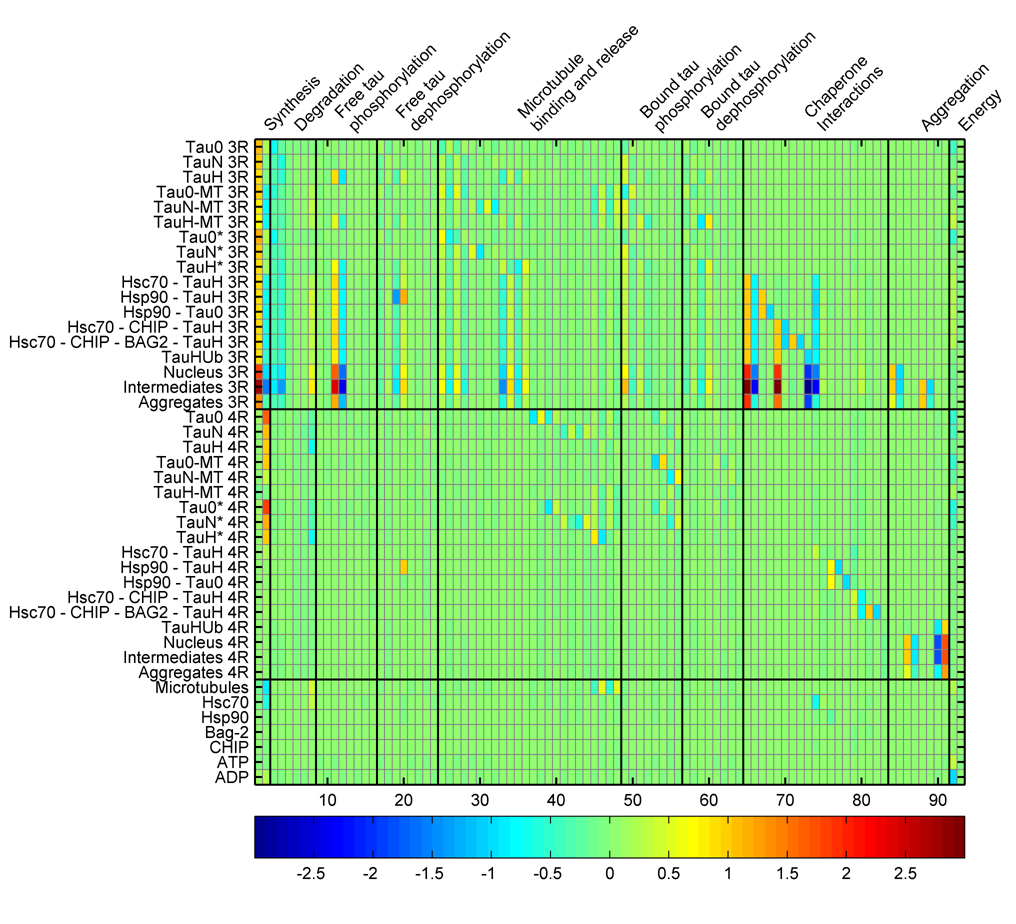

Supplement: Figure S6 — Relative, steady-state sensitivity for the aggregation-prone population of in silico neuron models. Median sensitivity coefficient at steady-state is shown for pairs of states (proteins) and parameters (rate constants). The parameters are grouped according to type. (0.80 MB TIF) [file pcbi.1000997.s009.tif]

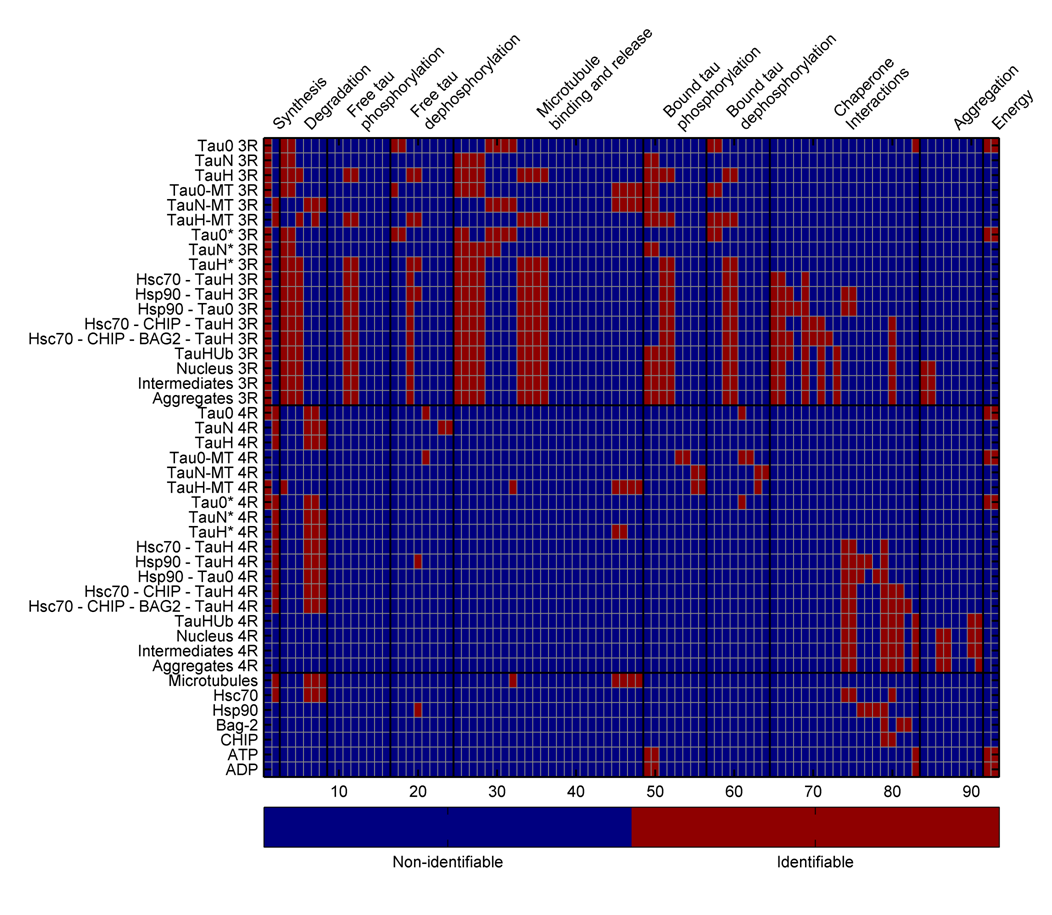

Supplement: Figure S7 — Identifiability of the sensitivity coefficients, as computed from the 95% confidence intervals. If the confidence interval spanned 0, the coefficient was labeled unidentifiable. (0.74 MB TIF) [file pcbi.1000997.s010.tif]

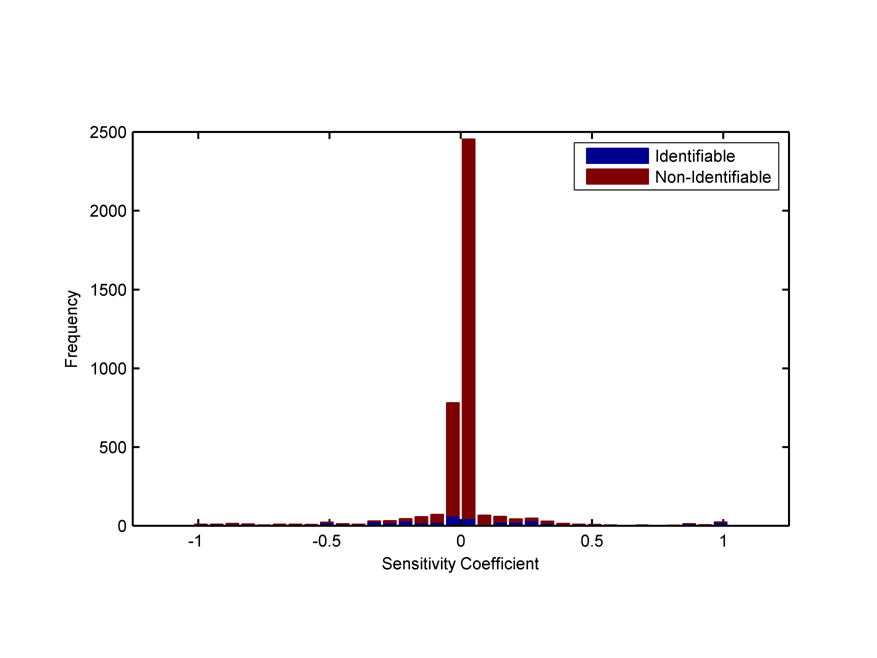

Supplement: Figure S8 — Distribution of the median sensitivity coefficients of the aggregation-prone population according to their identifiability and magnitude. (0.08 MB TIF) [file pcbi.1000997.s011.tif]
